# The Search for Antibacterial Inhibitors Targeting Cell Division Protein FtsZ at Its Nucleotide and Allosteric Binding Sites

**DOI:** 10.3390/biomedicines10081825

**Published:** 2022-07-28

**Authors:** José M. Andreu, Sonia Huecas, Lidia Araújo-Bazán, Henar Vázquez-Villa, Mar Martín-Fontecha

**Affiliations:** 1 Centro de Investigaciones Biológicas Margarita Salas, Consejo Superior de Investigaciones Científicas, Ramiro de Maeztu 9, 28040 Madrid, Spain; sonia@cib.csic.es (S.H.); laraujo@cib.csic.es (L.A.-B.); 2 Departamento de Química Orgánica, Facultad de Ciencias Químicas, Universidad Complutense de Madrid, Avda. Complutense s/n, 28040 Madrid, Spain; hvazquez@ucm.es; 3 Departamento de Química Orgánica, Facultad de Óptica y Optometría, Universidad Complutense de Madrid, Avda. Complutense s/n, 28040 Madrid, Spain

**Keywords:** antibiotics, bacterial division, FtsZ, nucleotide-replacing inhibitors, allosteric inhibitors

## Abstract

The global spread of bacterial antimicrobial resistance is associated to millions of deaths from bacterial infections per year, many of which were previously treatable. This, combined with slow antibiotic deployment, has created an urgent need for developing new antibiotics. A still clinically unexploited mode of action consists in suppressing bacterial cell division. FtsZ, an assembling GTPase, is the key protein organizing division in most bacteria and an attractive target for antibiotic discovery. Nevertheless, developing effective antibacterial inhibitors targeting FtsZ has proven challenging. Here we review our decade-long multidisciplinary research on small molecule inhibitors of bacterial division, in the context of global efforts to discover FtsZ-targeting antibiotics. We focus on methods to characterize synthetic inhibitors that either replace bound GTP from the FtsZ nucleotide binding pocket conserved across diverse bacteria or selectively bind into the allosteric site at the interdomain cleft of FtsZ from *Bacillus subtilis* and the pathogen *Staphylococcus aureus*. These approaches include phenotype screening combined with fluorescence polarization screens for ligands binding into each site, followed by detailed cytological profiling, and biochemical and structural studies. The results are analyzed to design an optimized workflow to identify effective FtsZ inhibitors, and new approaches for the discovery of FtsZ-targeting antibiotics are discussed.

## 1. Cell Division Protein FtsZ as an Antibacterial Target

Bacterial pathogens easily develop resistance to antibiotics used to treat infections. In some cases, strains can become resistant to all clinically employed antibiotics. This major threat to human health has not been met by the development of new antibiotics, meaning we face a post-antibiotic era where common bacterial infections are potentially untreatable and routine surgery or chemotherapy become too dangerous [1]. In fact, bacterial antimicrobial resistance (AMR) has been recently estimated to be associated to ~5 million deaths world-wide, of which ~1.3 million were directly attributable to AMR [2]. Among the top resistant pathogens, antibiotic resistant *Escherichia coli* was associated to ~0.8 million deaths, and methicillin-resistant *S. aureus* (MRSA) alone was estimated to cause more than 100,000 deaths and 3.5 million years of healthy life lost due to disability or premature death. There is thus an urgent need to discover new antibiotics and novel antibacterial targets.

Among the cellular processes essential for bacterial reproduction and infection, bacterial cell division [3] still remains clinically unexploited. The central protein organizing cell division in most bacteria is FtsZ, which together with membrane-tethering proteins forms a dynamic ring (Z-ring) at the division site, that recruits the other divisome proteins to coordinate cell wall remodeling and membrane constriction during cytokinesis [4,5,6,7]. FtsZ is an assembling GTPase whose structural core is shared by tubulin [8], the constituent of eukaryotic microtubules. Whereas tubulin is a classical target of numerous anticancer drugs, discovering new useful antibiotics targeting FtsZ remains a challenge. Over one hundred X-ray structures of tubulin-inhibitor complexes are available [9], but only a handful of FtsZ small-molecule complex structures have been reported to date [10,11,12,13,14,15,16], suggesting a lesser degree of druggability of FtsZ compared to tubulin. Nevertheless, considerable knowledge has been acquired about the structure, assembly dynamics, and function of FtsZ filaments and the Z-ring, the FtsZ partner proteins, and several small molecule chemotypes that specifically interact with FtsZ.

**Figure 1:**
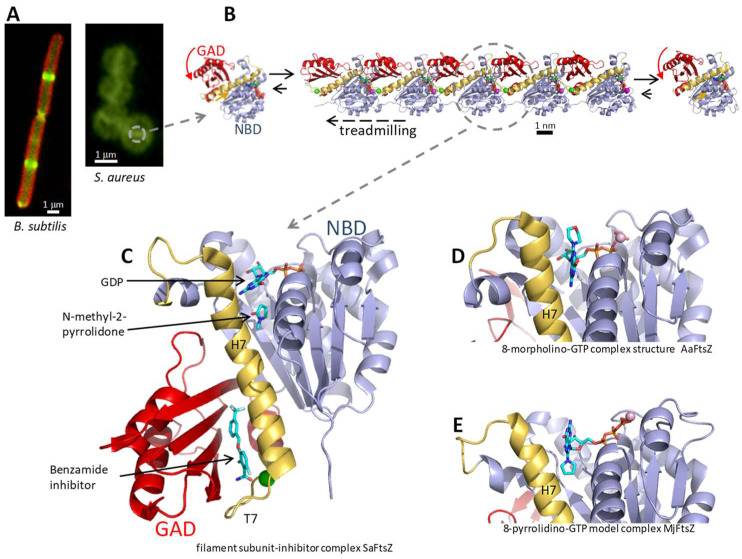
(**A**) Left, *B. subtilis* cells expressing FtsZ-GFP (green), with the membrane stained with FM4-64 (red); right, *S. aureus* cells treated with a fluorescent FtsZ probe (green, [25]) to visualize FtsZ rings. Scale bars: 1 μm. (**B**) Filament structure of FtsZ from *S. aureus*, represented by a pentamer (PDB 6RVN, [30]), with the treadmilling direction indicated by the arrow. One incoming monomer (left) and one dissociated monomer (right) are also shown. Scale bar: 1 nm. (**C**) Structure of one SaFtsZ filament subunit (cartoon representation). The N-terminal NBD is colored blue, the central helix H7 and its flanking loops yellow, and the C-terminal GAD red. The bound GDP nucleotide, a co-solvent molecule and one benzamide allosteric inhibitor are shown (sticks colored by atom type) [PDB 6YD6, [16]]. (**D**) AaFtsZ monomer in complex with 8-morpholino-GTP [PDB 2r7l, [10]]. (**E**) MjFtsZ monomer in complex with 8-pyrrolidino-GTP; NMR-based ligand model after docking and MD of the protein complex (adapted with permission from [48]; copyright 2013 American Chemical Society). All structural images displayed were prepared with PyMol [49].

The bacterial cytokinetic Z-ring (Figure 1A) is formed by condensates of FtsZ filaments that move circumferentially to guide correct septal peptidoglycan synthesis [17,18,19,20]. FtsZ is a protein assembly machine forming translocating filaments that grow from one end while shrinking from the other, in a process called treadmilling (Figure 1B). Treadmilling is driven by GTP hydrolysis, which takes place in the complete GTPase active site formed at the association interface between consecutive subunits in the filament, and triggers the disassembly of GDP-bound subunits at the shrinking end. The nucleotide-dependent interfacial interactions and the GTP hydrolysis mechanism have been recently revealed at atomic resolution by filament structures of SaFtsZ in different nucleotide states [21]. FtsZ cooperatively assembles into long single-stranded filaments, rather than forming short oligomers, which is made possible by conformational switching between a relaxed protein form (R) with low association affinity in monomers and a tense (T) high association affinity form in filaments [22,23,24,25,26]. The defined treadmilling direction (kinetic polarity) of FtsZ filaments is also enabled by the structural switch between T subunits inside filament and R monomers irrespective of the nucleotide bound, which creates structurally different encountering interfaces for association at each filament end [26,27].

The core structure of FtsZ consists of an N-terminal nucleotide-binding domain (NBD) and a C-terminal GTPase activation domain (GAD), joined by the long central helix H7 (Figure 1C). A variable unstructured C-terminal linker connects the FtsZ core to a binding motif for several of the FtsZ membrane tethering and partner proteins [5], whose interactions are also potential antibiotic targets [28]. FtsZ proteins spontaneously fold without requiring chaperones [29]. However, guanine nucleotide binding into its NBD pocket is required to stabilize the SaFtsZ core, which otherwise unfolds in the solution [30] and can thus be preferentially degraded by ADEP-activated ClpP protease [31]. Upon filament assembly, the interfacial GTPase site is completed as the GAD’s co-catalytic loop T7 inserts into the next subunit’s nucleotide binding pocket that adapts to the contact. The GAD itself rotates in the T-FtsZ conformation, opening a side interdomain cleft, flanked by helix H7 and the GAD´s beta-sheet, where the allosteric inhibitor PC190723 and related benzamides bind [11,12,13,16] (Figure 1C). The FtsZ inhibitor protein SulA [32] and peptide MciZ [33,34] bind the bottom of the GAD of FtsZ in the R-conformation, exerting monomer sequestering and filament capping activities.

FtsZ was soon recognized as an attractive antibacterial target [35,36] that was validated with the effective antistaphylococcal inhibitor PC190723 [11,37]. However, in a decade since our previous review [38], FtsZ inhibitors have not reached the clinic. It has been pointed out that many inhibitors reported in the literature were not really proven to specifically target FtsZ, slowing down progress in this field [39]. Currently, there are close to 200 publications with numerous compounds associated with FtsZ inhibition. The more recent reviews on FtsZ small molecule inhibitors include Refs. [40,41,42,43].

Among other approaches to targeting FtsZ, regulatory peptide MciZ inhibits FtsZ function across the *Bacillus* species and it is also recognized by FtsZ from *S. aureus* (SaFtsZ) [34]. However, conjugation with an effective bacterial membrane-penetrating peptide would be required for the delivery of MciZ into *S. aureus* cells. On the other hand, intracellular MRSA surviving antibiotic treatment within host cells is thought to represent an important pathogen reservoir in invasive and recurrent infections, which is experimentally eliminated by an antibody–antibiotic conjugate [44]. In addition to small molecule and peptide inhibitors, antisense peptide-nucleic acid mimetic polymers against the *ftsZ* gene conjugated to cell-penetrating peptides have been employed to suppress bacterial growth [45]. CRISPRi approaches can also be employed to control *ftsZ* transcription and thus bacterial division [46]. Targeted protein degradation approaches include FtsZ degradation by Clp cellular protease activated by an acyldepsipeptide (ADEP) antibiotic [47].

In this review we focus on the two main FtsZ binding sites for small molecules, the nucleotide site and the allosteric site, and the methods developed to screen and study specific antibacterial inhibitors targeting these sites.

## 2. The FtsZ Nucleotide Binding Site

### 2.1. Selective Inhibition of FtsZ Versus Tubulin by C8 Nucleotide Analogs

In view of the structural similarity of the FtsZ and tubulin enzymatic cores [8], a potential problem for FtsZ inhibitors could be cross-inhibiting human tubulin. However, both proteins share limited sequence identity, and it was soon realized that typical tubulin inhibitors do not inhibit FtsZ, whereas FtsZ inhibitors infrequently act on tubulin. A difference between the interfacial active sites of FtsZ and tubulin was demonstrated with GTP analogs bearing small hydrophobic substituents at the C8 position of the guanine ring, such as 8-methoxy-GTP and 8-Br-GTP, which bind with high affinity and selectively inhibit FtsZ polymerization in vitro, while promoting microtubule assembly and being substrates for tubulin GTPase [10]. The FtsZ assembly inhibitory activity of C8-GTP derivatives correlates with their binding affinity [10,48]. The crystal structure of thermophilic *Aquifex aeolicus* FtsZ (AaFtsZ) in complex with 8-morpholino-GTP [10] revealed a binding mode similar to GDP, with the morpholine ring oriented outward from the binding pocket towards the position of the next subunit in a protofilament (Figure 1D). A similar *anti* conformation of the glycosidic bond in the 8-morpholino-nucleotide bound to *B. subtilis* FtsZ (BsFtsZ) was deduced from NMR trNOESY analysis, whereas 8-pyrrolidino-nucleotide binds in the *syn* conformation, different from the natural nucleotide [48]. In this pose, the pyrrolidine ring is roughly in place of the guanine ring and the guanine ring points out from the binding pocket, as shown by a molecular dynamics (MD) model complex with thermophilic *Methanocaldococcus jannaschii* FtsZ (MjFtsZ, Figure 1E). Homology modeling BsFtsZ with bound C8-nucleotide analogs onto filaments of SaFtsZ (supported by a 80% protein sequence identity) showed that the inhibition of BsFtsZ assembly may be simply explained by steric clashes of the nucleotide analogs with the incoming FtsZ monomer [48]. The C8-GTP analogs were thus useful tools to demonstrate how to selectively inhibit FtsZ without inhibiting tubulin in vitro. However, they were devoid of antibacterial activity, possibly not passing the bacterial cell envelope. Initial attempts to design prodrugs based on C8-nucleotide analogs that would enter bacteria and be hydrolyzed in the cytosol to release C8-nucleotide inhibitors were unsuccessful [50]. Nevertheless, a different analog was recently reported, 3-amino-3-deoxyguanosine (ADG), which behaves as a prodrug mimic of GTP; ADG is phosphorylated to ADG-5′-triphosphate, a mimic of GTP that inhibits transcription and FtsZ-GFP localization to the Z-ring [51].

### 2.2. Fluorescent Nucleotide Analogs and Competition Assays for FtsZ Inhibitors

Among various fluorescent nucleotide conjugates widely employed to investigate nucleotide-binding proteins, we selected 2’/3’-*O*-(*N*-methylanthraniloyl)-GTP (*mant*-GTP), which bears the small fluorescent *mant* tag on the ribose ring, to probe the GTP-binding site of the nucleotide-devoid apo-MjFtsZ in monomers and filament polymers [52]. Both 2’- and 3’-substituted *mant*-GTP isomers are in equilibrium and both bind in the natural *anti*-glycosidic bond conformation, as deduced from NMR studies [53]. In both cases, the *mant* tag strongly interacts with FtsZ at an extension at the back of the GTP-binding pocket, as indicated by MD simulations. *Mant*-GTP can inhibit or induce FtsZ assembly and be hydrolyzed, depending of the FtsZ species employed [53].

On the other hand, a small water-filled cavity below the nucleotide-binding pocket is available for small ligand binding, such as co-solvent *N*-methyl-2-pyrrolydone in SaFtsZ crystals (Figure 1C) [13,16,30]. Together with the C-8 nucleotide analogs, these results underscore the plasticity of the FtsZ nucleotide binding site and support possible ligand extensions in the design of inhibitors.

The binding of *mant*-GTP to apo-FtsZ entails a reduction of the rotational diffusion of the probe that is detected by a marked increase of its fluorescence anisotropy. We employed this property to devise competition methods for any molecules able to replace the nucleotide in stable apo-MjFtsZ [54], in BsFtsZ [55,56], in apo-SaFtsZ, and also apo-FtsZ from *E. coli* (apo-EcFtsZ) stabilized by osmolytes [30]. These robust fluorescence polarization (FP) assays with stabilized apo-FtsZ monomers and a fluorescent GTP derivative can be manually performed or conveniently adapted for high-throughput screening (HTS) for nucleotide-replacing inhibitors. The assayed concentration of the compounds under study should be below their solubility limit. Insoluble compound precipitates are typically detected by interference from the scattering of polarized excitation light giving artifactually high anisotropy values. It is also important to exclude colloid-forming aggregators that may non-specifically bind and inactivate FtsZ [57]. Therefore, to determine ligand solubility and exclude FtsZ aggregation, we separately subject inhibitor and protein–inhibitor-*mant* GTP solutions to high-speed centrifugation, followed by measuring the inhibitor fraction and the probe fluorescence anisotropy remaining in the supernatant [55,56]. The *mant*-GTP FP competition method is thus useful for inhibitor screening and affinity measurements, although it is not meant to replace direct binding methods for inhibitor characterization, such as analytical co-sedimentation measurements [55] and isothermal titration calorimetry (ITC) when technically feasible [34].

### 2.3. Synthetic Nucleotide-Replacing FtsZ Inhibitors

**Figure 2:**
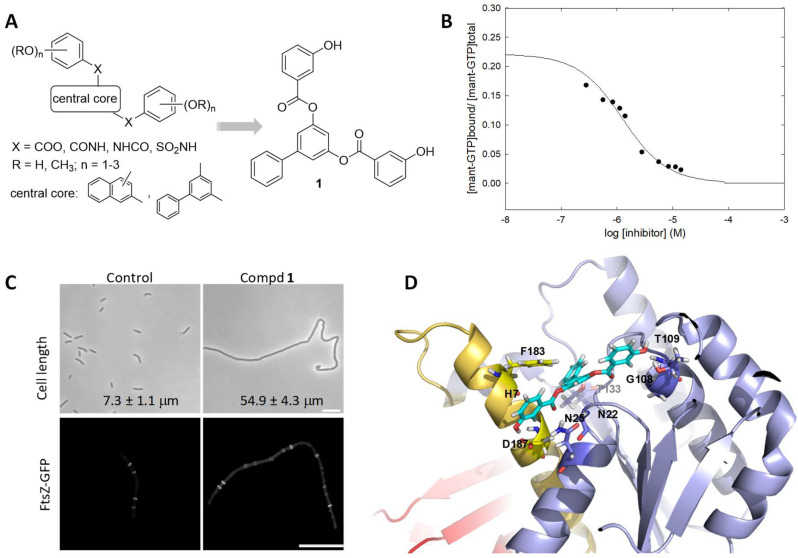
(**A**) General chemical structure of polyhydroxy aromatic GTP-replacing inhibitors and of the identified inhibitor **1** [55,56]. (**B**) Displacement of *mant*-GTP (1 μM) from BsFtsZ (0.9 μM binding sites) by compound **1**, measured by fluorescence anisotropy. The line is the least squares best-fitted competition curve (*K*_D_ = 0.5 μM). (**C**) Upper panel, cells of *B. subtilis* 168 treated with **1** (4 μM) for 3 h and observed by phase-contrast microscopy. Mean and standard error of cell length measurements are indicated. Lower panel, cellular localization of FtsZ-GFP in *B. subtilis* SU570 following growth for 1.5 h in the absence and presence of **1** (3 μM). Scale bars: 10 μm. (**D**) BsFtsZ-**1** model complex from docking and MD simulations. The ligand is shown in sticks represented with cyan carbon atoms. The amino acid residues that interact with the inhibitor and are shared key residues for nucleotide binding in the BsFtsZ-GDP complex [PBD 2RHL, [56]] are indicated and displayed in sticks. ((**B**–**D**) adapted with permission from [56]; copyright 2015 American Chemical Society).

We employed the *mant*-GTP FP assay to screen for GTP-replacing ligands among compounds from the literature [38], virtual screening (VS), and compounds selected from an in-house synthetic library by docking into the BsFtsZ GTP site. The latter yielded several polyhydroxy aromatic compounds that specifically bind to FtsZ, impair the localization of FtsZ to the Z-ring, inhibit *B. subtilis* cell division, and show antibacterial activity against clinical isolates of MRSA and levofloxacin-resistant *Enterococcus faecalis* [55]. A series of analogs with different aromatic scaffolds, linkers, and substitution patterns of the hydroxyl groups were then synthesized (Figure 2A) in iterative rounds guided by the binding affinity (*K*_D_ value in *mant*-GTP FP assays) and minimal division inhibitory concentration (MDC value on *B. subtilis*) below the MIC value. Among them, compound **1** binds into the GTP site with *K*_D_ = 0.5 μM (Figure 2B), impairs normal FtsZ assembly in vitro and FtsZ-GFP localization at the mid-cell (Figure 2C, 3 μM), and has a 7 μM MIC value on MRSA. In a BsFtsZ-**1** model complex, obtained by ligand docking and MD simulations, this inhibitor replaces GDP in several of its interactions with key residues in the nucleotide binding pocket (Figure 2D) [56]. Nevertheless, the affinity of these nucleotide-replacing inhibitors for FtsZ monomers is still lower than that of GTP, which is present at a high concentration in the bacterial cytosol.

It can be estimated that only a small fraction of the FtsZ molecules in a bacterial cell will be bound by compound **1** or its analogs. However, these inhibitors are able to effectively impair FtsZ assembly and inhibit cell division, which might be explained by the binding of the substoichiometric inhibitor by a few subunits perturbing the correct assembly of a FtsZ filament. Alternately the inhibitor-binding affinity for the nucleotide site in the filament association interface would have to be substantially larger than the affinity for monomers that is measured with the *mant*-GTP FP assay [55].

Interestingly, natural crysophaentins and synthetic hemi-crysophaentins are other examples of competitive inhibitors of nucleotide binding by FtsZ, endowed with antibacterial activity on *S. aureus*, as well as on permeable *E. coli envA1* cells, but not cytotoxic to mammalian cells [58,59]. Many of our nucleotide-replacing compounds selectively perturb FtsZ *vs* tubulin assembly, and their growth inhibitory concentrations on human cells are typically above those required for the inhibition of bacterial cell division [55,56]. It can thus be concluded that it is feasible to selectively inhibit bacterial division by targeting the FtsZ nucleotide binding site without cross-inhibiting mammalian cell tubulin. Curiously, despite the many microtubule targeting agents, to our knowledge, no tubulin nucleotide-replacing inhibitors have been discovered [9,60,61]. 

### 2.4. Antibacterial Activity of Nucleotide-Replacing FtsZ Inhibitors

**Figure 3:**
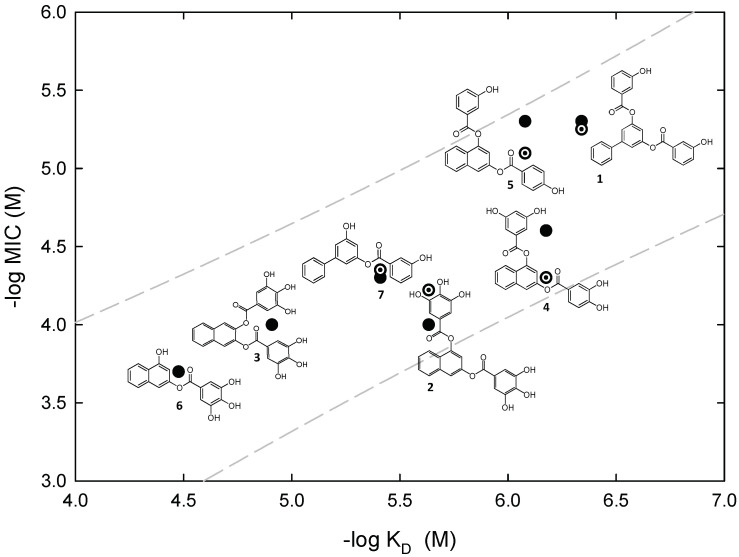
Correlation of antimicrobial activity (MIC) with binding affinity (*K*_D_) of inhibitor binding to the BsFtsZ nucleotide site. Data from representative analog compounds are shown together with their chemical structures. Filled circles, *B. subtilis* MIC; dotted circles, methicillin-resistant *S. aureus* MIC. The dashed lines are the 95% predictive intervals for *B. subtilis* MIC vs *K*_D_ values of 36 analogs (adapted with permission from [56]; copyright 2015 American Chemical Society).

The identified nucleotide-replacing inhibitors show activity on Gram-positive pathogens, particularly MRSA, over Gram-negative species [55,56,58], possibly related to an inability to cross the Gram-negative outer membrane [59] rather than to small differences in the FtsZ GTP-binding site across bacterial species. Interestingly, the antibacterial activity MIC values in our compound series correlate better with their affinity *K*_D_ values, than with FtsZ in vitro polymerization inhibition [56]. We favor the use of target affinity, rather than FtsZ polymerization or GTPase assays, as a predictor of antibacterial activity. Figure 3 shows several representative GTP-replacing inhibitors along the optimization process, on an activity–affinity log plot. It can be observed that the MIC values on MRSA (dotted circles) are within one factor of two of *B. subtilis* MIC values (filled circles). Further development of antibacterial nucleotide-replacing FtsZ inhibitors will, in our opinion, require enhancing chemical diversity by means of fragment screening, HTS, and new approaches discussed in Section 6.

## 3. The Filamenting Phenotype and the Cytological Profile of FtsZ Inhibitors

### 3.1. The Importance of Assessing Membrane Integrity

The inhibition of the divisomal protein function in bacilli results in characteristic cell elongation (filamentation), while cocci show an enlarged spherical size (ballooning). The name of the *ftsZ* gene comes from “filamenting temperature sensitive mutant Z” [62]. Thus, a convenient cell-based method to screen for FtsZ/divisomal inhibitors is the induction of a relevant filamenting phenotype, documented by images and cell length measurements (Figure 2C). However, similar filamentation effects may also be induced by a loss of the transmembrane potential, which results in FtsA and a secondary FtsZ release from the membrane [63]. In this context, several putative FtsZ inhibitors were shown to really act by impairing membrane potential and permeability [64]. Aminoglycoside antibiotics are known to have a second mode of action altering membrane integrity, but in the case of FtsZ, this would hamper target-based optimization. Therefore, any selected compound inducing bacterial cell filamentation should not be toxic at the MDC and must also be tested for membrane integrity (by exclusion of the DNA-binding fluorescent dye propidium iodide) and for membrane potential with a suitable probe (such as 3,3’-diethyloxacarbocyanine iodide, DiOC_2_) [65]. Whereas filamenting *B. subtilis* cells treated with our main GTP-replacing inhibitors, such as **1** (MDC 4 μM), had intact membranes and membrane potential, a few other analogs caused membrane lysis [56,65]. FtsZ inhibitors, following target-based optimization, might be combined with membrane depolarizing compounds, for the dual inhibition of cell division. On the other hand, several physiological mechanisms and proteins can inhibit FtsZ and block cell division, including the DNA damage SOS response with the expression of SulA, the nucleoid occlusion machinery, nutrient availability through moonlighting enzymes, sporulation through MciZ, and certain phage-encoded polypeptides [65] (and references therein).

### 3.2. The Cytological Profile of FtsZ Inhibitors

**Figure 4:**
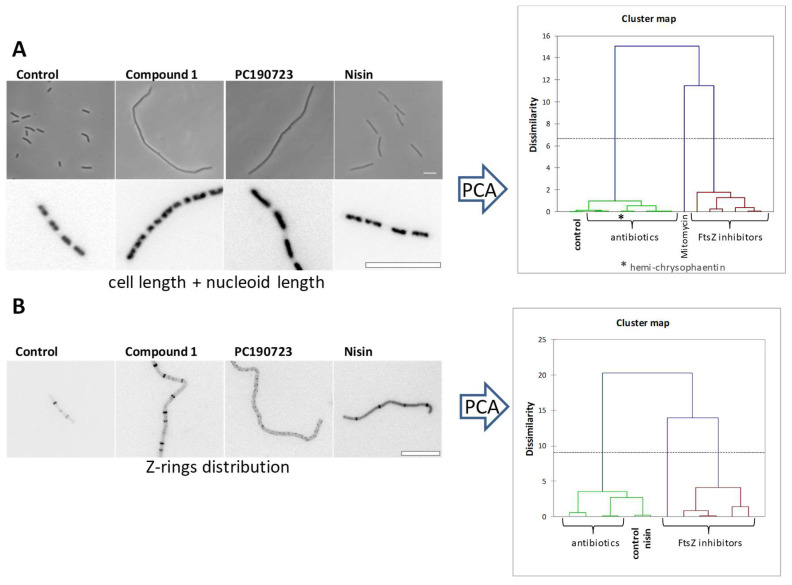
Cytological profile of antibacterial FtsZ inhibitors. Representative examples of *B. subtilis* cells observed upon treatment with **1** (3.5 μM), PC190723 (5.6 μM) and nisin (10 mg/L). (**A**) Undivided individual cells of *B. subtilis* 168 cells (phase contrast) and nucleoid morphology (DAPI). (**B**) Effects of FtsZ inhibitors and nisin on FtsZ subcellular localization on *B. subtilis* SU570 (FtsZ-GFP, contrast inverted). Scale bars: 10 μm. Right graphs, dendrograms obtained after a hierarchical cluster analysis based on values from a principal component analysis (PCA) using unweighted variables cell length and nucleoid length (upper graph) or Z-rings distribution (lower graph) [65].

In addition to cell filamentation measurements and FtsZ-GFP ring impairment observations (Figure 2C) typically employed for FtsZ inhibitor cell-based screening, cytological profiling methods for FtsZ targeting inhibitors in *B. subtilis* are available [65]. Bacterial cytological profiling uses quantitative fluorescence microscopy to rapidly identify the cellular mechanism of action of antibacterial agents, distinguishing different cellular pathways and targets [66]. Among several cellular effects of seven selected inhibitors binding to different FtsZ sites, principal component analysis and cluster maps of cell length combined with nucleoid length showed separate clusters for the FtsZ inhibitors (with one exception) and five known antibiotics with different mechanisms of action (Figure 4A). The distribution of distances between the remaining Z-rings in treated cells clearly distinguished six FtsZ inhibitors from known antibiotics and untreated control cells (Figure 4B). This study also revealed that exogenous synthetic polypeptide MciZ enters *B. subtilis* cells to effectively inhibit cell division [65], supporting the cytological profile approach for characterizing FtsZ inhibitors. MciZ modifies the treadmilling rate of FtsZ around the Z-ring in *B. subtilis* [17], and slows down *Bacillus* division, including pathogenic *B. cereus* and *B. anthracis* [34].

## 4. The FtsZ Interdomain Cleft

### 4.1. The Reference Antibacterial FtsZ Inhibitor PC190723

**Figure 5:**
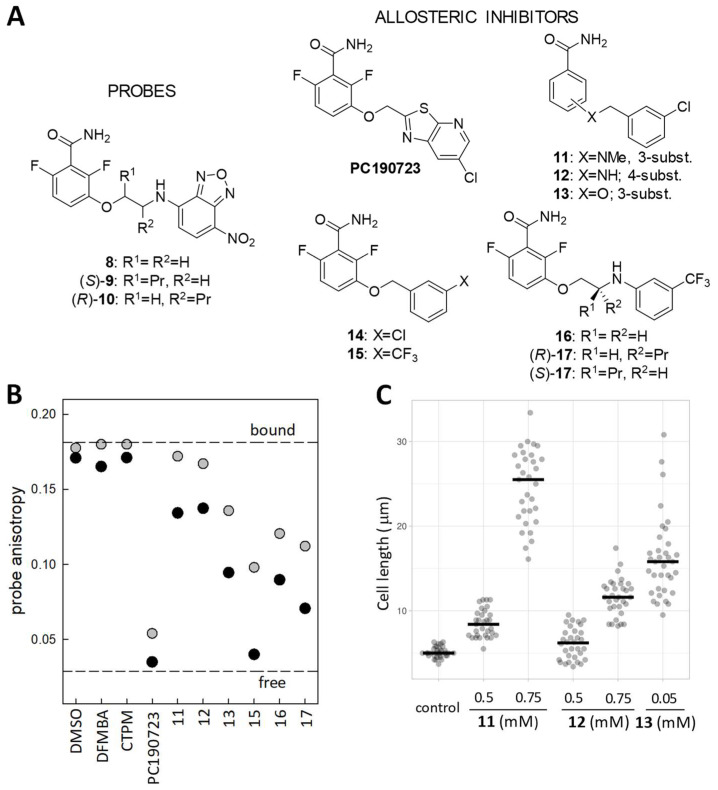
(**A**) Chemical structure of fluorescent probes and allosteric inhibitors targeting the FtsZ interdomain cleft. (**B**) Several examples from a florescence anisotropy screen for allosteric FtsZ inhibitors. The displacement of probe *(S)*-**9** (3 µM) from stabilized BsFtsZ polymers (8 µM binding sites) by two different concentrations of each compounds, 20 µM (gray dots) and 200 µM (black dots), is monitored. Control values with the solvent vehicle (DMSO), PC190723 and its 2,6-difluoro-3-methoxybenzamide (DFMBA) head and heterocyclic tail 6-(chloro [1,3] thiazolo [5,4-*b*] pyridin-2-yl)methanol (CTPM), are included. The dashed lines represent the fluorescence anisotropy of the probe partially bound by FtsZ polymers and of the free probe. (**C**) Cell length measurements of *B. subtilis* cells treated with compound **11**, **12**, and **13** for 3 h. Raw data plots with mean values (*n* ≥ 30) [16].

The small compound 3-methoxybenzamide was known to have weak antibacterial and filamentation activities, which are suppressed by mutations in the *ftsZ* gene of *B. subtilis* [67]. From this starting point, a large medicinal chemistry program guided by MIC and cell morphology [68] yielded the potent antistaphylococcal FtsZ difluorobenzamide inhibitor, PC190723 (Figure 5A) [11,37], which restores MRSA susceptibility to methicillin [11]. PC190723 is an inducer, instead of a suppressor, of FtsZ filament assembly and condensation [69] which makes FtsZ assemble into delocalized cellular *foci* rather than at the Z-ring [70]. By binding into the bottom of the open interdomain cleft, located between the GAD domain beta-sheet, helix H7, and loop T7 (Figure 1C), PC190723 stabilizes the open cleft T conformation of SaFtsZ and thus, filament formation, as shown by the crystal structure of the complex [11,12,71]. Unfortunately, PC190723 and its numerous benzamide analogs have not reached the clinic, possibly due to non-optimal pharmacological properties and to the relatively high frequency of resistance mutations in the *ftsZ* gene (FOR~10^−8^), although several improved derivatives are able to overcome certain resistance mutations [13,72] and reduce FOR in antibiotic combination therapy [73].

### 4.2. Fluorescent Probes for the FtsZ Interdomain Cleft and the Assembly Switch

In order to gain insight into the FtsZ interdomain cleft, we developed fluorescent PC190723 analogs based on the difluorobenzamide head, by replacing the heterocyclic tail with small fluorophores [25]. Among the synthesized compounds, several nitrobenzoxadiazole-based analogs were found to be suitable probes in terms of FtsZ affinity and fluorescent properties. The probes were selected for specific fluorescence anisotropy changes upon binding to polymers of BsFtsZ and SaFtsZ versus resistance mutants and Gram-negative FtsZ which cancelled binding. Probe **8** was biochemically characterized and shown to weakly label the Z-ring in bacterial cells, whereas at higher concentrations, it induced the characteristic cell division inhibition phenotype. The binding of probe **8** (*K*_D_ = 26 μM) was employed to monitor the opening and closing of the interdomain cleft during the BsFtsZ and SaFtsZ assembly–disassembly process, thus demonstrating the operation in solution of the FtsZ structural assembly switch [25], which had been inferred from the open cleft crystal structure of SaFtsZ filaments compared to other closed FtsZ monomer structures at that time. This was soon followed by the determination of closed structures of SaFtsZ monomers [13,26]. However, BsFtsZ or other filament structures have not been reported so far.

### 4.3. Fluorescence Polarization Assay for Allosteric FtsZ Inhibitors

Fluorescent benzamide probes with enhanced affinity (*S*)-**9** and (*R*)-**10** (Figure 5A; *K*_D_ values 8 μM and 2 μM, respectively) were designed and employed in FP competitive methods to screen for ligands binding into the FtsZ interdomain cleft and determine their binding affinity [16]. These methods use FtsZ polymers stabilized by mild crosslinking with glutaraldehyde, which do not disassemble upon nucleotide hydrolysis or by the action of assembly inhibitors binding elsewhere in the FtsZ molecule. Raw (*S*)-**9** probe fluorescence anisotropy values at two compound concentrations and solubility measurements can be employed to screen compounds binding into the FtsZ allosteric site, distinguishing low from higher affinity inhibitor candidates (Figure 5B). Combined with phenotypic screening (Figure 5C), even the action of relatively weak FtsZ-targeting compounds can be effectively detected, such as compound **11**, which is ~20-fold more active than 3-methoxybenzamide [16].

### 4.4. Enhancing the Affinity of Simplified Benzamide Inhibitors

**Figure 6:**
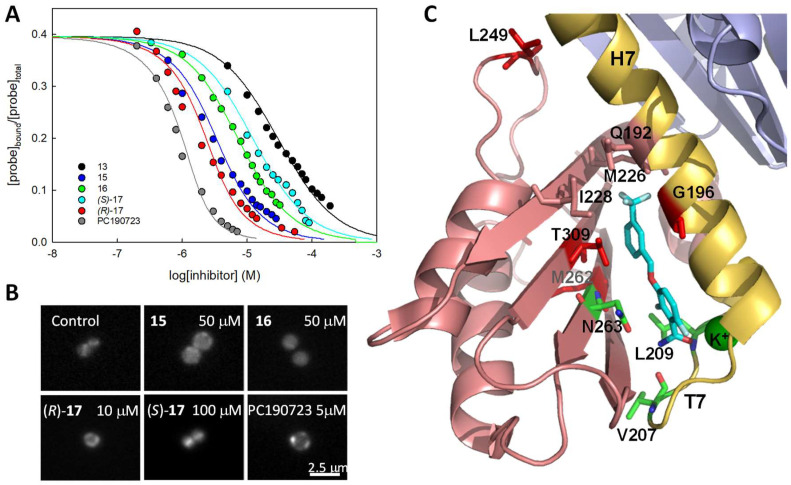
(**A**) Displacement curves of probe *(R)*-**10** (3 μM) from stabilized BsFtsZ polymers (2.7 μM binding sites) by synthetic allosteric inhibitors **13** (black), **15** (blue), **16** (green), *(R)*-**17** (red), *(S)*-**17** (cyan), and by PC190723 (gray). Lines correspond to the best-fitted competition curve in each case. (**B**) *S. aureus* cells expressing FtsZ-mCherry grown for 1.5 h in the absence (control) or presence of **15**, **16**, (*R*)-, *(S)*-**17**, and PC190723, observed by fluorescence microscopy. Scale bar: 2.5 μm. (**C**) Scheme of the structure of the SaFtsZ-**15** complex around the inhibitor (cyan)binding cleft. The positions of the MRSA inhibitor resistance mutations analyzed, G196S, L249V, M262I, and T309I, are indicated by the wild type side chains (red). Indicated as well are residues V207, L209, and N263 (green) that establish hydrogen bonds with the amide group of **15**. The T309 side chain has rotated to lodge the trifluoromethylphenyl ring between T309 and G196. The side chains of M226, I228, and Q192 (salmon) form a pore connecting the binding site to the upper part of the cleft. A K^+^ ion stabilizing loop T7 is also coordinated by the amide carbonyl of **15**. Not shown are additional residues making hydrophobic contacts with the inhibitor (PBD 6YD6; Table S4, Figures 6 and S6 in ref. [16]).

In a full competition assay using our FP method, the affinity of compounds replacing the probe (*R*)-**10** can be determined (Figure 6A). Combining affinity for BsFtsZ polymers and cell division inhibition activity on *B. subtilis*, a series of benzamide-based inhibitors with halogenated aryl tails (Figure 5A) were iteratively designed, reaching *K*_D_ and MDC values comparable to those of PC190723, while keeping a relatively simple chemical structure. Compound (*R*)-**17** has *K*_D_ = 0.2 μM and *B. subtilis* MDC = 1 μM. Derivatives **15**, **16**, *(R)*-**17**, and *(S)*-**17**, at a somewhat higher concentration, impair FtsZ-mCherry localization into the Z-ring (Figure 6B) and selectively inhibit the cell division and growth of *S. aureus* rather than the growth of human lung fibroblasts. FtsZ targeting was confirmed by analyzing spontaneous resistance mutations in MRSA, which predominantly mapped to the *ftsZ* gene, clustering around the FtsZ interdomain cleft [16]. Figure 6C shows the positions of the amino acids (in red) modified in the MRSA mutants, resistant to **15** around the interdomain cleft where the inhibitor binds.

### 4.5. Structural Insights into Allosteric Inhibitor Binding to S. aureus FtsZ

We have determined the high-resolution crystal structures of SaFtsZ in a complex with 2,6-difluoro-3-methoxybenzamide (DFMBA) and inhibitors **14** and **15**. In stark contrast, several other specifically binding compounds and the fluorescent probes did not show in the electron density maps [16], although they rendered stable MD model complexes [25]. Crystal structures of SaFtsZ in a complex with a different probe, TXA6101-Bodipy (BOPF), and with the inhibitors TXA6101 and TXA707 are also available [13,14]. A comparison of these structures shows that the protein interactions with the superimposable difluorobenzamide rings of each compound are conserved in all cases, including the amide group establishing hydrogen bonds to V207, L209, and N263, and coordinating a K^+^ ion bound by loop T7, as well as hydrophobic interactions made by the difluorobenzene ring. The halogenated phenyl moieties of **14** and **15** bind between T309 and G196, with their benzamide rings, adopting a flat geometry. The T309 residue acts as a gate, connecting with G196 in a closed conformation with DFMBA. Consequently, both G196 and T309 are among the positions modified by resistance mutations (Figure 6C). The M226 side chain constitutes another gate that gives access to a ligand-induced hydrophobic pocket for TXA707 and flexible inhibitor TXA6101 (not shown; [13]). The PC190723/**15** binding site connects through a pore, formed by the M226, I228, and G192 side chains, with a smaller pocket [16] and the upper large unfilled cavity above the interdomain cleft, which is flanked by the loop containing L249 (Figure 6C, surface representation in Figure 1 [16]). This interesting area, overlapping with the Taxol binding site of β-tubulin in microtubules [74], has not been targeted in FtsZ, but remains available for potential ligand extension [16,25].

### 4.6. Antibacterial Activity of Benzamide-Based Allosteric FtsZ Inhibitors

PC190723 and its analogs typically have narrow-spectrum antibacterial activity on several Gram-positive organisms, related to differences in the amino acid side chains lining the FtsZ interdomain cleft in other bacterial species [37]. Although this cleft is thought to open as well in T-state EcFtsZ filaments [26], two charged residue pairs have been proposed to form salt bridges between helix H7 and the C-terminal beta sheet, preventing cleft access to PC190723 in resistant species [75]. An allosteric modulator equivalent to PC190723 is thus missing for *E. coli* FtsZ, in addition to the general problem in crossing the outer membrane of Gram-negative bacteria [76], although the analog TXA6101 is active on *Klebsiella pneumoniae* [77].

**Figure 7:**
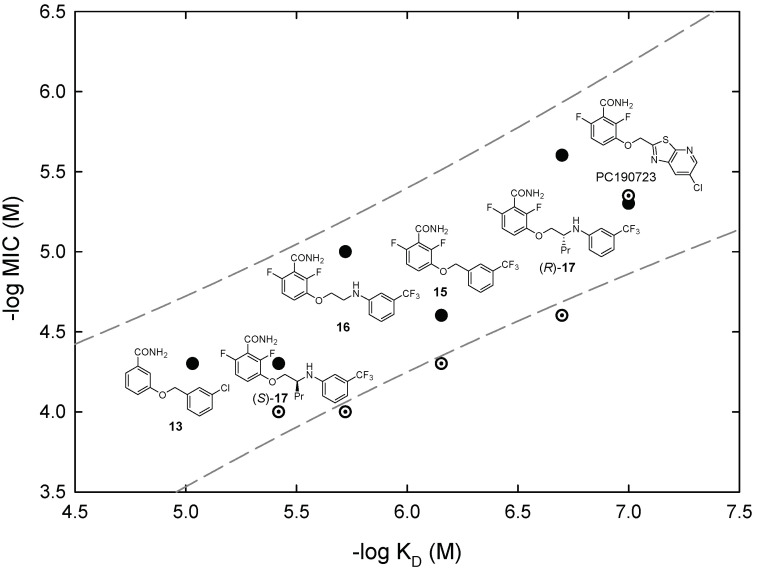
Correlation of antimicrobial activity (MIC) with binding affinity (*K*_D_) of inhibitor binding to the BsFtsZ benzamide site at the interdomain cleft. Data from representative analog compounds are shown together with their chemical structures. Filled circles, *B. subtilis* MIC; dotted circles, methicillin-resistant *S. aureus* MIC. The dashed lines are the 95% predictive intervals for *B. subtilis* MIC vs *K*_D_ values of 15 analogs [16].

Similarly to the nucleotide-replacing inhibitors, we found that the inhibition of bacterial growth correlates with the binding affinity of allosteric inhibitors [16], as shown for several representative inhibitors along the optimization process (Figure 7). On the other hand, changes in FtsZ GTPase activity are potentially misleading indicators of the activity of benzamide inhibitors, unless a complete analysis at different inhibitor and FtsZ concentrations is performed, due to their mixed FtsZ assembly promoting (GTPase activation) and GTPase modulatory effects [65]. In contrast with the nucleotide-replacing inhibitors, the antibacterial activity on the MRSA of our benzamide inhibitor series did not closely follow the activity of *B. subtilis*. The unexpectedly high MRSA MIC values of **17** (25 μM; PC190723 MIC = 5 μM) and **16** (100 μM) with an aminoethoxy linker are 10-fold larger than their MIC values on *B. subtilis* (Figure 7), perhaps due to permeability differences or to the action of staphylococcal efflux pumps. This shows the convenience of early testing for antibacterial activity on pathogenic species. Replacing the difluorobenzamide head of current inhibitors with other effective moieties remains a challenge. Alternately, allosteric FtsZ antibacterial inhibitors may be made more effective by ligand extensions predictively passing the pore to bind into the small pocket [78] and beyond, into the larger unfilled cavity between FtsZ domains (Figure 6), together with the optimization of their pharmacological properties.

## 5. Proposed Experimental Strategies for FtsZ Inhibitor Screening and Characterization

Following the development of FP binding and bacterial phenotype approaches during our search for FtsZ inhibitors, we would like to suggest two streamlined screening approaches for the identification and characterization of selective FtsZ inhibitors:

(A) To screen for FtsZ inhibitors, we currently favor the following workflow:
  (1) Phenotypic screening putative FtsZ inhibitors (1–10^2^ compounds) of *B. subtilis* and *E. coli* (cell filamentation), with the determination of the division inhibitory concentration (MDC) and membrane integrity.
    For HTS of large compound libraries, robotized phenotypic screening of *B. subtilis* or *E. coli* plate cultures, based on automatic image analysis of bacterial filamentation [79] may be implemented, if available.
  (2) FP determination of binding affinity (*K*_D_) to the nucleotide and allosteric binding sites of FtsZ and compound solubility limit determination.
  (3) Antibacterial activity (MIC) on pathogens and frequency of resistance (FOR) mutations of susceptible pathogens.
(B) As an alternate HTS route, we suggest a binding site-directed approach starting from a robotized FP plate screen of compound binding to the FtsZ nucleotide or allosteric sites, and compound solubility limit determination, followed by phenotypic analysis on *B. subtilis* and *E. coli* (filamentation), the determination of MDC and membrane integrity, and a final assessment of MIC and FOR in pathogens.

Each screening approach should be complemented by more detailed cytological profiling, selectivity, and biochemical and structural studies of selected hits, to be followed by the design of optimized analogs and their characterization. At this stage, pharmacokinetic studies should also be considered as a critical feature for the development of viable efficacious antibacterial drugs.

## 6. Outlook for Other Methods, VS, and Machine Learning Approaches for Selecting Potential Antibacterial FtsZ Inhibitors

Conventional HTS of natural extract and compound libraries for FtsZ inhibitors have historically yielded a few molecules that were not further developed (for example, viriditoxin [80], PC170942 [81]). Further screening of smaller focused and drug repurposing libraries still appears worthwhile. Larger areas of the chemical space currently available remain to be explored in natural sources and synthetic libraries, employing cell-based, target-binding, and computer-aided screens, in order to discover new FtsZ inhibitor chemotypes.

New cellular screens may be devised, for example, yeast cells expressing bacterial FtsZ-GFP fusions have been recently employed to monitor the effects of inhibitors on FtsZ assembly while assessing their toxicity of eukaryotic cells in a single-step microscopy screen. This approach [75] has revealed how PC190723 can bind into the interdomain cleft of FtsZ from Gram-negative *Helicobacter pylori,* as opposed to the case of EcFtsZ.

From a structural perspective, both the FtsZ nucleotide and the allosteric binding sites show annex cavities available for fragment binding (Figure 1 and Figure 6). This suggests the possibility of applying HT X-ray crystallographic screening of fragment libraries and further structure-guided fragment-based approaches [82] to develop new FtsZ inhibitors. Several NMR methods may be also applied to search for FtsZ-binding fragments, including ligand-based fluorine NMR screening [83].

Virtual screening (VS) may be employed to expand the chemical diversity of potential inhibitors, as well as to narrow down experimental inhibitor screening. However, early docking-based VS campaigns for FtsZ inhibitors were frequently met by a lack of success, or produced compounds that were not further developed [for example, Refs. [54,84,85]]. More recently, we selected a series of 41 compounds by flexible docking VS with ICM [86] of 4 million compounds from the Zinc library [87,88] against the BsFtsZ nucleotide-binding site. These were experimentally selected for specific binding in place of *mant*-GTP and the inhibiting division of *B. subtilis* cells, followed by studying 43 well-docking chemically similar compounds from MolPort and Mcule commercial libraries [89]. However, the more active compounds also impaired the membrane potential [unpublished results], which hampered further optimization and underscores the importance of testing membrane integrity. We also screened the Zinc library against an ensemble of seven open conformations of the SaFtsZ PC190723 binding site, selecting seven benzamide and thirty-eight non-benzamide compounds. Among them, five compounds were weak competitors of the specific fluorescent probe **8** for binding to BsFtsZ polymers, but only one benzamide derivative was a weak inhibitor of *B. subtilis* cell division [90]. Complementary to structure-based VS, the chemical features of well-characterized FtsZ small molecule ligands could be employed in ligand-based VS for FtsZ inhibitors.

Artificial intelligence (AI), employing a neural network trained with experimental *E. coli* growth inhibition screening, has been successfully employed to discover new antibiotics [91]. AI drug discovery companies are producing a growing number of discovery and preclinical stage compounds in several therapeutic areas, some of which are in clinical trials [92]. Currently, AI-enabled VS permits screen ultra-large, billion-molecule chemical libraries, combining structural docking with ligand-based prediction [93]. It is thus conceivable that FtsZ-focused AI approaches employing learning data sets from new cell-based and target binding screens, combined with available FtsZ-ligand complex structures and ADMET scoring, may yield new antibacterial FtsZ inhibitors.

## 7. Conclusions

Three decades after the discovery of the central role of FtsZ in bacterial division [4], FtsZ remains a challenging target for discovering new antibiotics. It may be asked whether the profound basic knowledge of the FtsZ structural assembly mechanism and function that has been achieved can be effectively employed to discover new antibiotics. To further address this problem, we propose combining target-specific screening methods such as those reviewed here with a knowledge-based design and new AI approaches in the quest for FtsZ-targeting antibiotics.

## Data Availability

No new data were created or analyzed in this study. Data sharing is not applicable to this article.

## References

[1] Kwon J.H., Powderly W.G. (2021). The post-antibiotic era is here. Science.

[2] Murray C.J.L., Ikuta K.S., Sharara F., Swetschinski L., Robles Aguilar G., Gray A., Han C., Bisignano C., Rao P., Wool E. (2022). Global burden of bacterial antimicrobial resistance in 2019: A systematic analysis. Lancet.

[3] den Blaauwen T., Andreu J.M., Monasterio O. (2014). Bacterial cell division proteins as antibiotic targets. Bioorganic Chem..

[4] Bi E., Lutkenhaus J. (1991). FtsZ ring structure associated with division in Escherichia coli. Nature.

[5] McQuillen R., Xiao J. (2020). Insights into the structure, function, and dynamics of the bacterial cytokinetic FtsZ-ring. Annu. Rev. Biophys..

[6] Barrows J.M., Goley E.D. (2021). FtsZ dynamics in bacterial division: What, how, and why?. Curr. Opin. Cell Biol..

[7] Levin P.A., Janakiraman A., Slauch J.M. (2021). Localization, assembly, and activation of the Escherichia coli cell division machinery. EcoSal Plus.

[8] Nogales E., Downing K.H., Amos L.A., Lowe J. (1998). Tubulin and FtsZ form a distinct family of GTPases. Nat. Struct. Biol..

[9] Steinmetz M.O., Prota A.E. (2018). Microtubule-targeting agents: Strategies to hijack the cytoskeleton. Trends Cell Biol..

[10] Läppchen T., Pinas V.A., Hartog A.F., Koomen G.J., Schaffner-Barbero C., Andreu J.M., Trambaiolo D., Lowe J., Juhem A., Popov A.V. (2008). Probing FtsZ and tubulin with C8-substituted GTP analogs reveals differences in their nucleotide binding sites. Chem. Biol..

[11] Tan C.M., Therien A.G., Lu J., Lee S.H., Caron A., Gill C.J., Lebeau-Jacob C., Benton-Perdomo L., Monteiro J.M., Pereira P.M. (2012). Restoring methicillin-resistant Staphylococcus aureus susceptibility to beta-lactam antibiotics. Sci. Transl. Med..

[12] Matsui T., Yamane J., Mogi N., Yamaguchi H., Takemoto H., Yao M., Tanaka I. (2012). Structural reorganization of the bacterial cell-division protein FtsZ from Staphylococcus aureus. Acta Cryst. Sect. D-Biol. Crystallogr..

[13] Fujita J., Maeda Y., Mizohata E., Inoue T., Kaul M., Parhi A.K., LaVoie E.J., Pilch D.S., Matsumura H. (2017). Structural flexibility of an inhibitor overcomes drug resistance mutations in Staphylococcus aureus FtsZ. ACS Chem. Biol..

[14] Ferrer-González E., Fujita J., Yoshizawa T., Nelson J.M., Pilch A.J., Hillman E., Ozawa M., Kuroda N., Al-Tameemi H.M., Boyd J.M. (2019). Structure-guided design of a fluorescent probe for the visualization of FtsZ in clinically important gram-positive and gram-negative bacterial pathogens. Sci. Rep..

[15] Alnami A., Norton R.S., Pena H.P., Haider S., Kozielski F. (2021). Conformational flexibility of a highly conserved helix controls cryptic pocket formation in FtsZ. J. Mol. Biol..

[16] Huecas S., Araújo-Bazán L., Ruiz F.M., Ruiz-Ávila L.B., Martínez R.F., Escobar-Peña A., Artola M., Vázquez-Villa H., Martín-Fontecha M., Fernández-Tornero C. (2021). Targeting the FtsZ allosteric binding site with a novel fluorescence polarization screen, cytological and structural approaches for antibacterial discovery. J. Med. Chem..

[17] Bisson-Filho Alexandre W., Hsu Y.-P., Squyres Georgia R., Kuru E., Wu F., Jukes C., Sun Y., Dekker C., Holden S., VanNieuwenhze Michael S. (2017). Treadmilling by FtsZ filaments drives peptidoglycan synthesis and bacterial cell division. Science.

[18] Yang X., Lyu Z., Miguel A., McQuillen R., Huang K.C., Xiao J. (2017). GTPase activity-coupled treadmilling of the bacterial tubulin FtsZ organizes septal cell wall synthesis. Science.

[19] Monteiro J.M., Pereira A.R., Reichmann N.T., Saraiva B.M., Fernandes P.B., Veiga H., Tavares A.C., Santos M., Ferreira M.T., Macario V. (2018). Peptidoglycan synthesis drives an FtsZ-treadmilling-independent step of cytokinesis. Nature.

[20] Squyres G.R., Holmes M.J., Barger S.R., Pennycook B.R., Ryan J., Yan V.T., Garner E.C. (2021). Single-molecule imaging reveals that Z-ring condensation is essential for cell division in Bacillus subtilis. Nat. Microbiol..

[21] Ruiz F.M., Huecas S., Santos-Aledo A., Prim E.A., Andreu J.M., Fernández-Tornero C. (2022). FtsZ filament structures in different nucleotide states reveal the mechanism of assembly dynamics. PLoS Biol..

[22] Huecas S., Llorca O., Boskovic J., Martin-Benito J., Valpuesta J.M., Andreu J.M. (2008). Energetics and geometry of FtsZ polymers: Nucleated self-assembly of single protofilaments. Biophys. J..

[23] Miraldi E.R., Thomas P.J., Romberg L. (2008). Allosteric models for cooperative polymerization of linear polymers. Biophys. J..

[24] Fujita J., Harada R., Maeda Y., Saito Y., Mizohata E., Inoue T., Shigeta Y., Matsumura H. (2017). Identification of the key interactions in structural transition pathway of FtsZ from Staphylococcus aureus. J. Struct. Biol..

[25] Artola M., Ruiz-Avila L.B., Ramirez-Aportela E., Martinez R.F., Araujo-Bazán L., Vázquez-Villa H., Martín-Fontecha M., Oliva M.A., Martín-Galiano A.J., Chacón P. (2017). The structural assembly switch of cell division protein FtsZ probed with fluorescent allosteric inhibitors. Chem. Sci..

[26] Wagstaff J.M., Tsim M., Oliva M.A., García-Sanchez A., Kureisaite-Ciziene D., Andreu J.M., Löwe J. (2017). A polymerisation-associated conformational switch in FtsZ. mBio.

[27] Du S., Pichoff S., Kruse K., Lutkenhaus J. (2018). FtsZ filaments have the opposite kinetic polarity of microtubules. Proc. Natl. Acad. Sci. USA.

[28] Zorrilla S., Monterroso B., Robles-Ramos M.-Á., Margolin W., Rivas G. (2021). FtsZ interactions and biomolecular condensates as potential targets for new antibiotics. Antibiotics.

[29] Andreu J.M., Oliva M.A., Monasterio O. (2002). Reversible unfolding of FtsZ cell division proteins from archaea and bacteria. Comparison with eukaryotic tubulin folding and assembly. J. Biol. Chem..

[30] Huecas S., Canosa-Valls A.J., Araújo-Bazán L., Ruiz F.M., Laurents D.V., Fernández-Tornero C., Andreu J.M. (2020). Nucleotide-induced folding of cell division protein FtsZ from Staphylococcus aureus. FEBS J..

[31] Silber N., Pan S., Schäkermann S., Mayer C., Brötz-Oesterhelt H., Sass P., Søgaard-Andersen L. (2020). Cell division protein FtsZ is unfolded for N-terminal degradation by antibiotic-activated ClpP. mBio.

[32] Cordell S.C., Robinson E.J., Lowe J. (2003). Crystal structure of the SOS cell division inhibitor SulA and in complex with FtsZ. Proc. Natl. Acad. Sci. USA.

[33] Bisson-Filho A.W., Discola K.F., Castellen P., Blasios V., Martins A., Sforca M.L., Garcia W., Zeri A.C., Erickson H.P., Dessen A. (2015). FtsZ filament capping by MciZ, a developmental regulator of bacterial division. Proc. Natl. Acad. Sci. USA.

[34] Araujo-Bazan L., Huecas S., Valle J., Andreu D., Andreu J.M. (2019). Synthetic developmental regulator MciZ targets FtsZ across Bacillus species and inhibits bacterial division. Mol. Microbiol..

[35] Vollmer W. (2006). The prokaryotic cytoskeleton: A putative target for inhibitors and antibiotics?. Appl. Microbiol. Biotechnol..

[36] Lock R.L., Harry E.J. (2008). Cell-division inhibitors: New insights for future antibiotics. Nat. Rev. Drug. Discov..

[37] Haydon D.J., Stokes N.R., Ure R., Galbraith G., Bennett J.M., Brown D.R., Baker P.J., Barynin V.V., Rice D.W., Sedelnikova S.E. (2008). An inhibitor of FtsZ with potent and selective anti-staphylococcal activity. Science.

[38] Schaffner-Barbero C., Martin-Fontecha M., Chacon P., Andreu J.M. (2012). Targeting the assembly of bacterial cell division protein FtsZ with small molecules. ACS Chem. Biol..

[39] Kusuma K.D., Payne M., Ung A.T., Bottomley A.L., Harry E.J. (2019). FtsZ as an Antibacterial Target: Status and Guidelines for Progressing This Avenue. ACS Infect. Dis..

[40] Silber N., Matos de Opitz C.L., Mayer C., Sass P. (2020). Cell division protein FtsZ: From structure and mechanism to antibiotic target. Future Microbiol..

[41] Casiraghi A., Suigo L., Valoti E., Straniero V. (2020). Targeting bacterial cell division: A binding site-centered approach to the most promising inhibitors of the essential protein FtsZ. Antibiotics.

[42] Han H., Wang Z., Li T., Teng D., Mao R., Hao Y., Yang N., Wang X., Wang J. (2021). Recent progress of bacterial FtsZ inhibitors with a focus on peptides. FEBS J..

[43] Pradhan P., Margolin W., Beuria T.K. (2021). Targeting the achilles heel of FtsZ: The interdomain cleft. Front. Microbiol..

[44] Lehar S.M., Pillow T., Xu M., Staben L., Kajihara K.K., Vandlen R., DePalatis L., Raab H., Hazenbos W.L., Hiroshi Morisaki J. (2015). Novel antibody–antibiotic conjugate eliminates intracellular S. aureus. Nature.

[45] Nejad A.J., Shahrokhi N., Nielsen P.E. (2021). Targeting of the essential acpP, ftsZ, and rne genes in carbapenem-resistant Acinetobacter baumannii by antisense PNA precision antibacterials. Biomedicines.

[46] Mückl A., Schwarz-Schilling M., Fischer K., Simmel F.C. (2018). Filamentation and restoration of normal growth in Escherichia coli using a combined CRISPRi sgRNA/antisense RNA approach. PLoS ONE.

[47] Sass P., Josten M., Famulla K., Schiffer G., Sahl H.-G., Hamoen L., Brötz-Oesterhelt H. (2011). Antibiotic acyldepsipeptides activate ClpP peptidase to degrade the cell division protein FtsZ. Proc. Natl. Acad. Sci. USA.

[48] Marcelo F., Huecas S., Ruiz-Avila L.B., Canada F.J., Perona A., Poveda A., Martin-Santamaria S., Morreale A., Jimenez-Barbero J., Andreu J.M. (2013). Interactions of bacterial cell division protein FtsZ with C8-substituted guanine nucleotide inhibitors. A combined NMR, biochemical and molecular modeling perspective. J. Am. Chem. Soc..

[49] Schrodinger, LLC. (2015). The PyMOL Molecular Graphics System.

[50] Lappchen T. (2007). Synthesis of GTP Analogues and Evaluation of Their Effect on the Antibiotic Target FtsZ and Its Eukaryotic Homologue Tubulin. Ph.D. Thesis.

[51] Shahsavari N., Wang B., Imai Y., Mori M., Son S., Liang L., Böhringer N., Manuse S., Gates Michael F., Morrissette M. (2022). A silent operon of photorhabdus luminescens encodes a prodrug mimic of GTP. mBio.

[52] Huecas S., Schaffner-Barbero C., Garcia W., Yebenes H., Palacios J.M., Diaz J.F., Menendez M., Andreu J.M. (2007). The interactions of cell division protein FtsZ with guanine nucleotides. J. Biol. Chem..

[53] Huecas S., Marcelo F., Perona A., Ruiz-Avila L.B., Morreale A., Canada F.J., Jimenez-Barbero J., Andreu J.M. (2015). Beyond a Fluorescent Probe: Inhibition of Cell Division Protein FtsZ by mant-GTP Elucidated by NMR and Biochemical Approaches. ACS Chem. Biol..

[54] Schaffner-Barbero C., Gil-Redondo R., Ruiz-Avila L.B., Huecas S., Lappchen T., den Blaauwen T., Diaz J.F., Morreale A., Andreu J.M. (2010). Insights into nucleotide recognition by cell division protein FtsZ from a mant-GTP competition assay and molecular dynamics. Biochemistry.

[55] Ruiz-Avila L.B., Huecas S., Artola M., Vergonos A., Ramirez-Aportela E., Cercenado E., Barasoain I., Vazquez-Villa H., Martin-Fontecha M., Chacon P. (2013). Synthetic Inhibitors of Bacterial Cell Division Targeting the GTP-Binding Site of FtsZ. ACS Chem. Biol..

[56] Artola M., Ruiz-Avila L.B., Vergoñós A., Huecas S., Araujo-Bazán L., Martín-Fontecha M., Vázquez-Villa H., Turrado C., Ramírez-Aportela E., Hoegl A. (2015). Effective GTP-replacing FtsZ inhibitors and antibacterial mechanism of action. ACS Chem. Biol..

[57] Anderson D.E., Kim M.B., Moore J.T., O’Brien T.E., Sorto N.A., Grove C.I., Lackner L.L., Ames J.B., Shaw J.T. (2012). Comparison of small molecule inhibitors of the bacterial cell division protein FtsZ and identification of a reliable cross-species inhibitor. ACS Chem. Biol..

[58] Plaza A., Keffer J.L., Bifulco G., Lloyd J.R., Bewley C.A. (2010). Chrysophaentins A-H, antibacterial bisdiarylbutene macrocycles that inhibit the bacterial cell division protein FtsZ. J. Am. Chem. Soc..

[59] Keffer J.L., Huecas S., Hammill J.T., Wipf P., Andreu J.M., Bewley C.A. (2013). Chrysophaentins are competitive inhibitors of FtsZ and inhibit Z-ring formation in live bacteria. Bioorg. Med. Chem..

[60] Matthew S., Chen Q.-Y., Ratnayake R., Fermaintt Charles S., Lucena-Agell D., Bonato F., Prota Andrea E., Lim Seok T., Wang X., Díaz J.F. (2021). Gatorbulin-1, a distinct cyclodepsipeptide chemotype, targets a seventh tubulin pharmacological site. Proc. Natl. Acad. Sci. USA.

[61] Mühlethaler T., Gioia D., Prota A.E., Sharpe M.E., Cavalli A., Steinmetz M.O. (2021). Comprehensive analysis of binding sites in tubulin. Angew. Chem. Int. Ed..

[62] Lutkenhaus J.F., Wolf-Watz H., Donachie W.D. (1980). Organization of genes in the ftsA-envA region of the Escherichia coli genetic map and identification of a new fts locus (ftsZ). J. Bacteriol..

[63] Strahl H., Hamoen L.W. (2010). Membrane potential is important for bacterial cell division. Proc. Natl. Acad. Sci. USA.

[64] Foss M.H., Eun Y.J., Grove C.I., Pauw D.A., Sorto N.A., Rensvold J.W., Pagliarini D.J., Shaw J.T., Weibel D.B. (2013). Inhibitors of bacterial tubulin target bacterial membranes. MedChemComm.

[65] Araujo-Bazan L., Ruiz-Avila L.B., Andreu D., Huecas S., Andreu J.M. (2016). Cytological profile of antibacterial FtsZ inhibitors and synthetic peptide MciZ. Front. Microbiol..

[66] Nonejuie P., Burkart M., Pogliano K., Pogliano J. (2013). Bacterial cytological profiling rapidly identifies the cellular pathways targeted by antibacterial molecules. Proc. Natl. Acad. Sci. USA.

[67] Ohashi Y., Chijiiwa Y., Suzuki K., Takahashi K., Nanamiya H., Sato T., Hosoya Y., Ochi K., Kawamura F. (1999). The lethal effect of a benzamide derivative, 3-methoxybenzamide, can be suppressed by mutations within a cell division gene, ftsZ, in Bacillus subtilis. J. Bacteriol..

[68] Haydon D.J., Bennett J.M., Brown D., Collins I., Galbraith G., Lancett P., Macdonald R., Stokes N.R., Chauhan P.K., Sutariya J.K. (2010). Creating an Antibacterial with in Vivo Efficacy: Synthesis and Characterization of Potent Inhibitors of the Bacterial Cell Division Protein FtsZ with Improved Pharmaceutical Properties. J. Med. Chem..

[69] Andreu J.M., Schaffner-Barbero C., Huecas S., Alonso D., Lopez-Rodriguez M.L., Ruiz-Avila L.B., Nunez-Ramirez R., Llorca O., Martin-Galiano A.J. (2010). The antibacterial cell division inhibitor PC190723 is an FtsZ polymer-stabilizing agent that induces filament assembly and condensation. J. Biol. Chem..

[70] Adams D.W., Wu L.J., Czaplewski L.G., Errington J. (2011). Multiple effects of benzamide antibiotics on FtsZ function. Mol. Microbiol..

[71] Elsen N.L., Lu J., Parthasarathy G., Reid J.C., Sharma S., Soisson S.M., Lumb K.J. (2012). Mechanism of action of the cell-division inhibitor PC190723: Modulation of FtsZ assembly cooperativity. J. Am. Chem. Soc..

[72] Stokes N.R., Baker N., Bennett J.M., Berry J., Collins I., Czaplewski L.G., Logan A., Macdonald R., Macleod L., Peasley H. (2013). An improved small-molecule inhibitor of FtsZ with superior in vitro potency, drug-like properties, and in vivo efficacy. Antimicrob. Agents Chemother..

[73] Kaul M., Mark L., Parhi A.K., LaVoie E.J., Pilch D.S. (2016). Combining the FtsZ-targeting prodrug TXA709 and the cephalosporin cefdinir confers synergy and reduces the frequency of resistance in methicillin-resistant Staphylococcus aureus. Antimicrob. Agents Chemother..

[74] Kellogg E.H., Hejab N.M.A., Howes S., Northcote P., Miller J.H., Díaz J.F., Downing K.H., Nogales E. (2017). Insights into the distinct mechanisms of action of taxane and non-taxane microtubule stabilizers from cryo-EM structures. J. Mol. Biol..

[75] Sharma A.K., Poddar S.M., Chakraborty J., Nayak B.S., Kalathil S., Mitra N., Pananghat G., Srinivasan R. (2022). A salt bridge mediated resistance mechanism to FtsZ inhibitor PC190723 revealed by a single step cell-based screen. bioRxiv.

[76] Richter M.F., Drown B.S., Riley A.P., Garcia A., Shirai T., Svec R.L., Hergenrother P.J. (2017). Predictive compound accumulation rules yield a broad-spectrum antibiotic. Nature.

[77] Rosado-Lugo J.D., Sun Y., Banerjee A., Cao Y., Datta P., Zhang Y., Yuan Y., Parhi A.K. (2022). Evaluation of 2,6-difluoro-3-(oxazol-2-ylmethoxy)benzamide chemotypes as Gram-negative FtsZ inhibitors. J. Antibiot..

[78] Straniero V., Sebastián-Pérez V., Suigo L., Margolin W., Casiraghi A., Hrast M., Zanotto C., Zdovc I., Radaelli A., Valoti E. (2021). Computational design and development of benzodioxane-benzamides as potent inhibitors of FtsZ by exploring the hydrophobic subpocket. Antibiotics.

[79] Fredborg M., Rosenvinge F.S., Spillum E., Kroghsbo S., Wang M., Sondergaard T.E. (2015). Automated image analysis for quantification of filamentous bacteria. BMC Microbiol..

[80] Wang J., Galgoci A., Kodali S., Herath K.B., Jayasuriya H., Dorso K., Vicente F., González A., Cully D., Bramhill D. (2003). Discovery of a small molecule that inhibits cell division by blocking FtsZ, a novel therapeutic target of tntibiotics. J. Biol. Chem..

[81] Stokes N.R., Sievers J., Barker S., Bennett J.M., Brown D.R., Collins I., Errington V.M., Foulger D., Hall M., Halsey R. (2005). Novel inhibitors of bacterial cytokinesis identified by a cell-based antibiotic screening assay. J. Biol. Chem..

[82] Charoensutthivarakul S., Thomas S.E., Curran A., Brown K.P., Belardinelli J.M., Whitehouse A.J., Acebrón-García-de-Eulate M., Sangan J., Gramani S.G., Jackson M. (2022). Development of inhibitors of SAICAR synthetase (PurC) from Mycobacterium abscessus using a fragment-based approach. ACS Infect. Dis..

[83] Dalvit C., Vulpetti A. (2019). Ligand-based fluorine NMR screening: Principles and applications in drug discovery projects. J. Med. Chem..

[84] Chan F.-Y., Sun N., Neves M.A.C., Lam P.C.-H., Chung W.-H., Wong L.-K., Chow H.-Y., Ma D.-L., Chan P.-H., Leung Y.-C. (2013). Identification of a new class of FtsZ inhibitors by structure-based design and in vitro screening. J. Chem. Inf. Model..

[85] Sun N., Chan F.-Y., Lu Y.-J., Neves M.A.C., Lui H.-K., Wang Y., Chow K.-Y., Chan K.-F., Yan S.-C., Leung Y.-C. (2014). Rational design of berberine-based FtsZ inhibitors with broad-spectrum antibacterial activity. PLoS ONE.

[86] Totrov M., Abagyan R. (1997). Flexible protein-ligand docking by global energy optimization in internal coordinates. Proteins.

[87] Irwin J.J., Shoichet B.K. (2005). ZINC − A free database of commercially available compounds for virtual screening. J. Chem. Inf. Model..

[88] ZINC15. https://zinc.docking.org.

[89] Vergoñós-Tomás A. (2017). Inhibidores de la Proteína de División Celular Bacteriana FtsZ Dirigidos al Sitio de Unión del Nucleótido. Ph.D. Thesis.

[90] Ramirez-Aportela E. (2017). Dinámica de los Filamentos de FtsZ y Búsqueda Racional de Inhibidores Sintéticos con Actividad Antibacteriana. Ph.D. Thesis.

[91] Stokes J.M., Yang K., Swanson K., Jin W., Cubillos-Ruiz A., Donghia N.M., MacNair C.R., French S., Carfrae L.A., Bloom-Ackermann Z. (2020). A deep learning approach to antibiotic discovery. Cell.

[92] Jayatunga M.K.P., Xie W., Ruder L., Schulze U., Meier C. (2022). AI in small-molecule drug discovery: A coming wave?. Nat. Rev. Drug Discov..

[93] Gentile F., Yaacoub J.C., Gleave J., Fernandez M., Ton A.-T., Ban F., Stern A., Cherkasov A. (2022). Artificial intelligence–enabled virtual screening of ultra-large chemical libraries with deep docking. Nat. Protoc..

